# Oridonin inhibits tumor angiogenesis and induces vessel normalization in experimental colon cancer

**DOI:** 10.7150/jca.55929

**Published:** 2021-04-02

**Authors:** Jing Zhou, Yaocheng Li, Xuejing Shi, Shulan Hao, Fupeng Zhang, Zhi Guo, Yu Gao, Hao Guo, Likun Liu

**Affiliations:** 1 Department of Oncology, Shanxi Province Academy of Traditional Chinese Medicine, Shanxi Province Hospital of Traditional Chinese Medicine, Taiyuan, Shanxi 030012, China.; 2 Department of Anesthesiology, Shanxi provincial people's Hospital, Taiyuan, Shanxi 030000, China.

**Keywords:** colon cancer, oridonin, angiogenesis, vascular normalization, JAK2/STAT3

## Abstract

**Purpose:** Tumor blood vessels exhibit morphological and functional aberrancies. Its maturity and functionality are closely associated with colon cancer progression and therapeutic efficacy. The direct evidence proving whether oridonin (ORI) has vascular normalization promoting effect from which combination therapies will benefit is still lacking.

**Methods:** We established a subcutaneous xenograft model of human colon cancer. The animals were divided into the Control and ORI-treated groups. Immunohistochemical analysis and TUNEL staining was applied to evaluate the proliferation, apoptosis and angiogenesis. Western blot analysis was employed to characterize the angiogenesis-related factors and JAK2/STAT3 signaling. Then, vascular normalization and macrophage reprogramming were assessed by immunofluorescence analysis.

**Results:** The results showed that ORI obviously reduced tumor growth, diminished the numbers of Ki67^+^ cells and CD31^+^ microvessel density, while increased the numbers of TUNEL^+^ cells. The expression levels of VEGF and bFGF proteins were dramatically down-regulated while the angiostatin and endostatin levels were increased in the ORI-treated group. Moreover, ORI therapy remarkably promoted the pericyte coverage of tumor vessels from days 5 to 10, with the highest pericyte coverage rate occurred at day 7. In the time window of vascular normalization, hypoxia of the tumor microenvironment was improved by ORI, the expression of HIF-1a was downregulated. Moreover, CD206^+^ macrophage cells were diminished in the ORI-treated group. These anticancer effects of ORI maybe partly mediated by suppressing JAK2/STAT3 signaling pathway.

**Conclusions:** These results highlight the potential effect of ORI on anti-angiogenesis and inducing vessel normalization roles of ORI, and probably provide optimum time point for the ORI therapy in conjunction with the chemoradiotherapy or immunotherapy.

## Introduction

Colon cancer is one of the most common malignancies cancers observed worldwide. Although current therapeutic strategies significantly improve the prognosis, the 5-year overall survival rate is still relatively low 1,2. The discovery of novel therapeutic targets against colon cancer is urgently needed. Fortunately, exploring therapeutic drugs from the library of herbal medicines offers excellent colon cancer treatment 3. Oridonin (ORI), a natural compound extracted from Chinese herb Rabdosia Rubescens and/or related species, has attracted increasing attention due to the potential pharmacological anti-tumor effects on colon cancer 4-6. Nevertheless, based on the complicated pathological mechanisms of colon cancer, the details concerning the underlying molecular mechanism of ORI's anti-cancer effects still need further elucidation.

The aggressive nature of colon cancer appears to be closely connected with the degree of angiogenesis 7,8. An increasing number of anti-angiogenesis therapy has been extensively developed for cancer treatment, effectively suppressing tumor growth 9-11. However, accumulating data reveal that anti-angiogenesis therapies destroy the delivery of oxygen and therapeutics dependent vasculatures can simultaneously potentiate the therapeutic effects of radiation and chemotherapy 12-14. This seems paradoxical. Fortunately, the proposal of the conception of “vascular normalization” can reasonably explain this synergistic phenomenon 15. Distinguished from the normal ones, tumor blood vessels exhibit morphological and functional aberrancies. The abnormal vessels lead to a hostile tumor microenvironment (TME) characterized by interstitial hypertension, hypoxia and acidosis. This induced TME facilitates metastatic potential of tumor cells and simultaneously impedes immune cell infiltration into the tumor. Anti-angiogenic therapy can cause a temporary reversion of tumor vessels towards a normalized structure and function. These improvements can promote the delivery of therapeutic drugs and oxygen into the tumor. It plays a critical role in cancer progression 16. Previous studies have demonstrated that ORI has a valid anti-cancer response on colon cancer through anti-angiogenic effects 17. However, the underlying mechanisms remained unclear. In addition, direct evidence proving whether ORI has a vascular normalization effect from which combination therapies could benefit is still lacking.

Janus kinase 2 (JAK2)/signal transducer and activator of transcription 3 (STAT3) signaling pathway is believed as a crucial link implicated in invasion, survival, growth and angiogenesis of cancer cells 18. Activated JAK could phosphorylate the tyrosine of STAT. The phosphorylated STAT can be transferred into the nucleus to bind to the specific promoter to induce the expression of target mRNAs. Previous studies have suggested that JAK2/STAT3 pathway participates in the regulation of angiogenesis, and blockade of the JAK2/STAT3 pathway could inhibit tumor growth 19. We inferred that JAK2/STAT3 pathway inhibition might participate in ORI's therapeutic effects. Herein, we established a subcutaneous xenograft tumor model of colon cancer with ORI administration, and unraveled the possible mechanisms underlying this effect.

## Materials and Methods

### Cell culture

Human HCT116 colon cancer cell was purchased from the Shanghai Institutes for Biological Sciences (China). Cells were cultured in Dulbecco's modified Eagle's medium in 5% CO_2_ at 37 °C. The cells were supplemented with 10% fetal bovine serum (Gibco, USA), with 1% penicillin/streptomycin.

### Animal preparation and establishment of the xenograft tumor model

Male BALB/c nude mice (4-6 weeks old) were purchased from SPF (Beijing) Biotechnology Co.,Ltd. (Beijing, China). Animals were housed in a temperature-controlled room with 12 hours light/dark cycling under pathogen-free conditions, and had free access to food and water. The experimental protocol was established, and performed according to the guidelines and was approved by the Institutional Animal Care and Use Committee of Shanxi Province Academy of Traditional Chinese Medicine. HCT116 Cells (2×10^6^/100 µL of PBS) were injected subcutaneously into the right flank of the mice. Tumor volume (TV) was calculated using the following formula: TV (mm^3^) = a×b^2^/2 (a: length; b: width). The experiments were initiated when the tumor volume reached 50-100 mm^3^.

### Experimental design and treatment groups

All animals were randomly assigned to the following experiments as described.

The first set - animals were randomly divided into (1) Control group: mice in this group received vehicle only; (2) ORI group: mice were intragastric administered with ORI (40 mg/kg) once daily. The size of subcutaneous tumors was measured every three days using calipers. At three weeks after treatment, tumor xenografts were removed.

The second set - to explore the promoting vessel normalization effects of ORI, partial animals in Control and ORI group were sacrificed at days (d) 3, 5, 7, 10 d or 12 after treatment.

### TdT-mediated dUTP nick end labeling (TUNEL) staining

Paraffin-embedded tumor tissue sections were deparaffinized, rehydrated. To assess cellular apoptosis, the sections were incubated with proteinase K working solution for 30 min at 37 °C. After washing three times with PBS buffer, then incubated with 50 μL of TUNEL reaction mixture, then add a lid and incubated for 2 hours at 37 °C in the dark. Then, the slides were incubated with 100 μL stopping buffer for 10 minutes and then rinsed in PBS three times. DAPI was applied to detect the nuclei. Images were observed via a fluorescence microscope, and the percentage of the dUTP-positive cells was detected.

### Immunohistochemical analysis

Sections were immersed in 3% hydrogen peroxide for 15 min. Nonspecific antigen blocking was performed in 2% bovine serum albumin (BSA) for 1 hour. Sections were then incubated overnight at 4 °C with rabbit anti-Ki67 (1:200, Abcam), rabbit anti-CD31 (1:200, Bioworld), or rabbit anti-HIF-1a (1:100, Boster), then incubated with biotinylated anti-rabbit IgG (1:100, Santa Cruz) for 1 hour at 37 °C, with the avidin-biotinperoxidase complex (1:100, Vector Laboratories) for 1 hour at 37 °C. Immunoreactivity was visualized with diaminobenzidine staining and imaged under a microscope.

To explore the promoting vessel normalization effects of ORI, we performed immunofluorescence double labeling. Sections were incubated in rabbit anti-CD31 (1:200, Bioworld), with mouse anti-a-SMA (1:100, Boster) for 1 hour at 37 °C. For macrophage polarization assessment, sections were incubated in mouse anti-CD206 (1:100, Santa Cruz) for 1 hour at 37 °C. Antibodies were then used: the fluorescein 488-conjugated donkey anti-rabbit antibody (1:1000, Jackson Immunoresearch, USA), or Cy3-conjugated sheep anti-mouse antibody (1:1000, Jackson Immunoresearch). The visual data were scanned using a fluorescence microscope.

### Western blot

Tissues were homogenized in RIPA lysis buffer with protease inhibitor. The homogenate was centrifuged at 12,000 rpm for 30 min at 4 °C. Protein concentration was assayed with the BCA method. Protein was loaded and subjected to electrophoresis in 10% SDS-PAGE gels and transferred onto PVDF membranes. The membranes were then blocked in 5% BSA for 2 hour. The membranes were incubated with primary antibodies as follows: rabbit anti-JAK2 (1:1000, Cell Signaling Technology), or rabbit anti-p-JAK2 (1:1000, Cell Signaling Technology), or mouse anti-STAT3 (1:1000, Cell Signaling Technology), or mouse anti-p-STAT3 (1:2000, Cell Signaling Technology), or rabbit anti-β-actin (1:6000, Abcam), or rabbit anti-VEGF (1:1000, Servicebio), or rabbit anti-bFGF (1:1000, Bioworld), or rabbit anti-angiostatin (1:1000, Bioworld), or rabbit anti- endostatin (1:1000, Boster), with gentle shaking at 4 °C overnight. Then, horseradish peroxidaseconjugated goat anti-mouse IgG (1:50000, Proteintech) or goat anti-rabbit IgG (1:50000, Proteintech) secondary antibodies were incubated with the membranes for 2 hours at room temperature. The immunopositive bands were visualized using an enhanced chemiluminescent substrate (Thermo Fisher) and Bio-Rad ChemiDoc XRS digital documentation system. The amount of protein expression is presented relative to the levels of β-actin.

### Statistical analysis

The results of all experiments are expressed as the mean ± standard deviation (SD). Comparisons between two groups were assessed by Student's t-test, and a *p*-value < 0.05 was considered statistically significant.

## Results

### ORI inhibited tumor growth in colon cancer model

**Figure 1:**
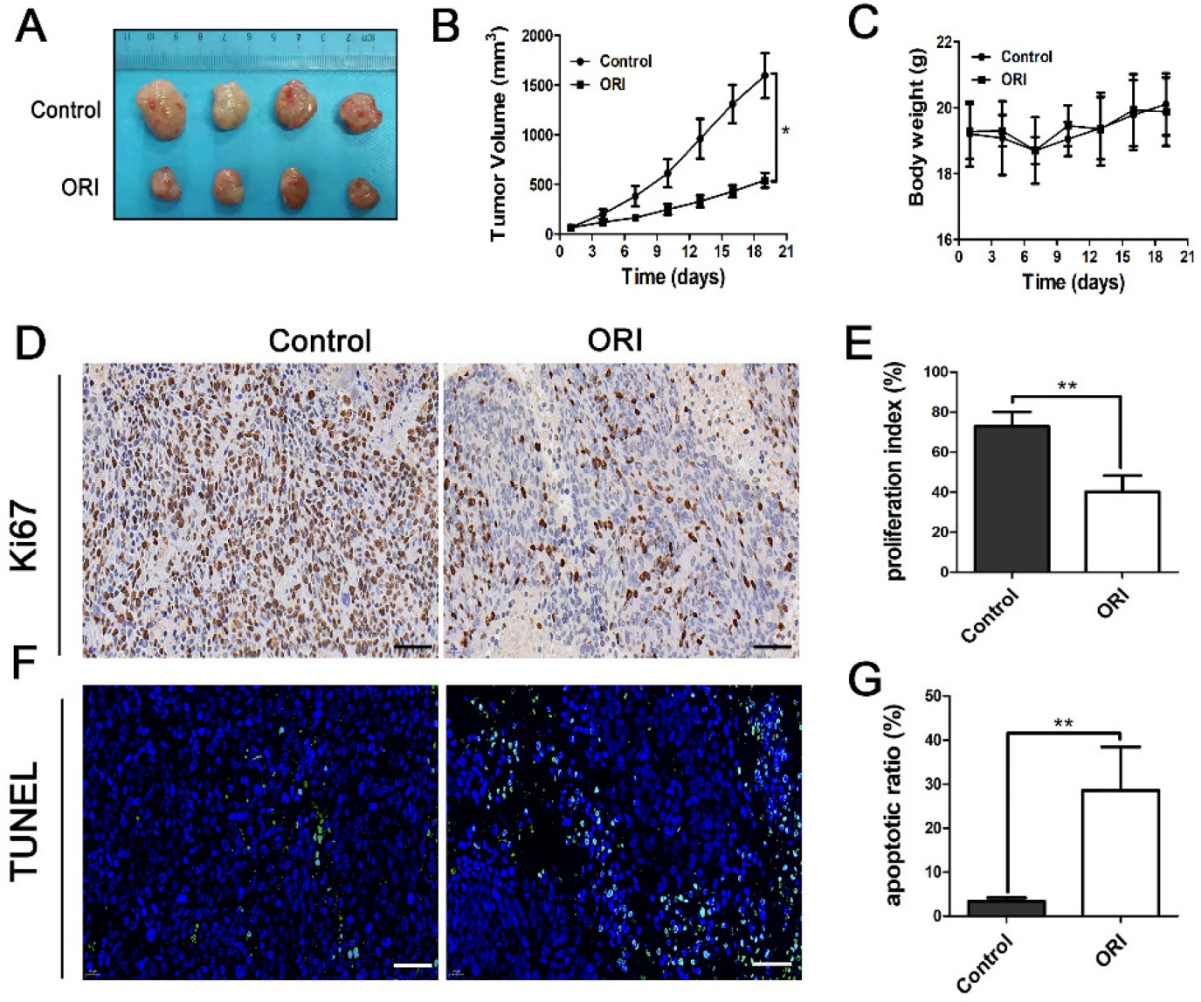
** ORI inhibited tumor growth, proliferation and induced apoptosis in colon cancer.** (A) Photo of HCT116 tumors resected from mice of Control and ORI groups. (B) The tumor volume in the ORI-treated group was significantly smaller than the control group. (C) The body weight of the two groups has no significant difference. (D) Representative images of immunohistochemical analysis of Ki67 in the tumor (× 400, scale bar =50 µm). (E) The expression of Ki67 was remarkably diminished in the ORI-treated group. (F) Representative images of TUNEL analysis (× 400, scale bar =50 µm). (G) ORI resulted in significantly more TUNEL^+^ cells in the tumor. *p < 0.05, **p < 0.01.

A colon cancer HCT116 cell xenograft nude mouse model was applied to explore ORI's anti-tumor effect* in vivo*. We found that the ORI-treated group's tumor volume was significantly smaller than the Control group (Figure 1A, 1B). The results demonstrated the tumor-suppressed capacity and potential use of ORI in colon cancer treatment. Furthermore, comparing ORI-treated group with the Control group, there was no significant intergroup difference in body weight during the therapy (Figure 1C), indicating that the ORI treatment had no significant toxicity in mice.

### ORI inhibited the proliferation and induced apoptosis in colon cancer

We investigated the effects of ORI on tumor cell proliferation and apoptosis. Immunohistochemical analysis showed the expression of Ki67 was remarkably diminished in the ORI-treated group, indicated that ORI depressed proliferation phenotype (Figure 1D and 1E). Tumor cell apoptosis which was determined by TUNEL expression assay was used. It showed that only a basal level of apoptotic cell death could be observed in the control group. At the same time, ORI resulted in significantly more TUNEL^+^ cells in the tumor (Figure 1F and 1G).

### ORI inhibited angiogenesis and influenced the pro-angiogenic factors and anti-angiogenic factors expression in colon cancer

**Figure 2:**
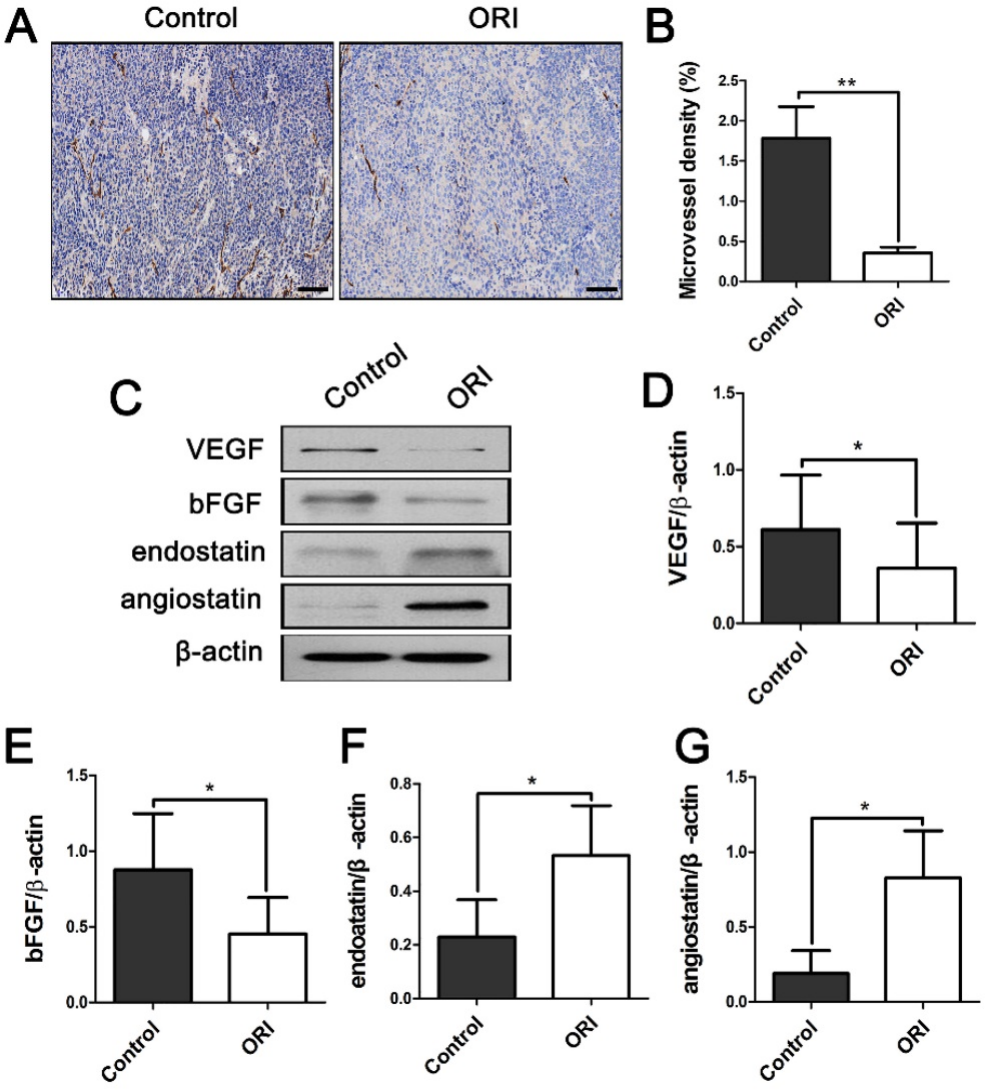
** ORI inhibited angiogenesis of colon cancer.** (A) Representative images of immunohistochemical analysis of tumor microvessels (×200, scale bar =100 µm). (B) CD31^+^ MVD was dramatically suppressed in ORI- treated group compared to the Control group. (C) Representative Western blot expressions of VEGF, bFGF, angiostatin and endostatin. (D and E) The levels of VEGF and bFGF were dramatically down-regulated in the ORI-treated group, while (F and G) angiostatin and endostatin were increased by treatment with ORI. *p < 0.05, **p < 0.01.

To explore whether ORI can affect the angiogenesis of tumors, tissue sections were stained with antibodies against CD31 to detect the microvessel density (MVD). Pro-angiogenic and anti-angiogenic factors present vital regulatory effects on tumor angiogenesis. In addition, the expression levels of pro-angiogenic related factors vascular endothelial growth factor (VEGF), basic fibroblast growth factor (bFGF) and anti-angiogenic factors angiostatin, endostatin were observed. CD31^+^ MVD was dramatically suppressed in ORI-treated group compared to the Control group (Figure 2A and 2B). As it shown, VEGF and bFGF (pro-angiogenic factors) were dramatically down-regulated in the ORI-treated group (Figure 2C, 2D and 2E). In contrast, angiostatin and endostatin (anti-angiogenic factors) were increased by treatment with ORI (Figure 2C, 2F and 2G).

### ORI promoted vessel normalization in colon cancer

**Figure 3:**
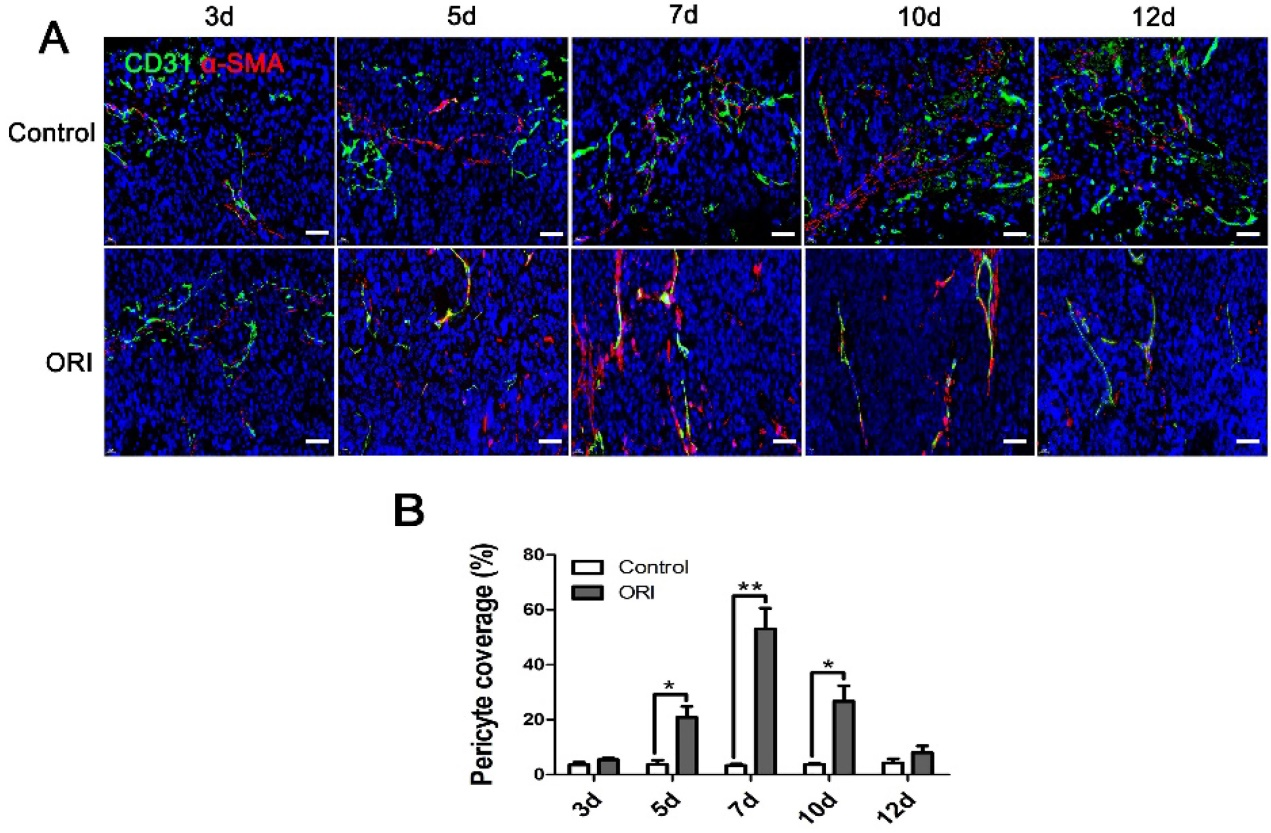
** ORI promoted vessel normalization in colon cancer.** (A) Tumor vessels were immunostained for CD31 (green) and pericytes for α-SMA (red). (×400, scale bar =50 µm). (B) The significantly increased perivascular cell coverage in the ORI group was observed from day 5 until day 10 compared to the Control group, the highest of perivascular cell coverage for ORI occurred on day 7.

The neovasculature in tumors usually exhibits immaturity as indicated by the lack of pericyte coverage around the vascular endothelial cells. To explore the ORI-induced vessel normalization, the structure and function of vessels were investigated. CD31^+^/α-SMA^+^ double immunofluorescent labeling was detected in a series of days (3 d, 5 d, 7 d, 10 d and 12 d) after ORI treatment. In Figure 3A and 3B, the significantly increased pericyte coverage rate in the ORI group were observed from day 5 until day 10 when compared with the Control group. It revealed that after ORI treatment, the vessels were more mature and normalized. The highest pericyte coverage rate for ORI occurred on day 7. The pericyte coverage rate on day 12 had no significant differences with the Control which suggested the shutting down of the vessel normalization window.

**Figure 4:**
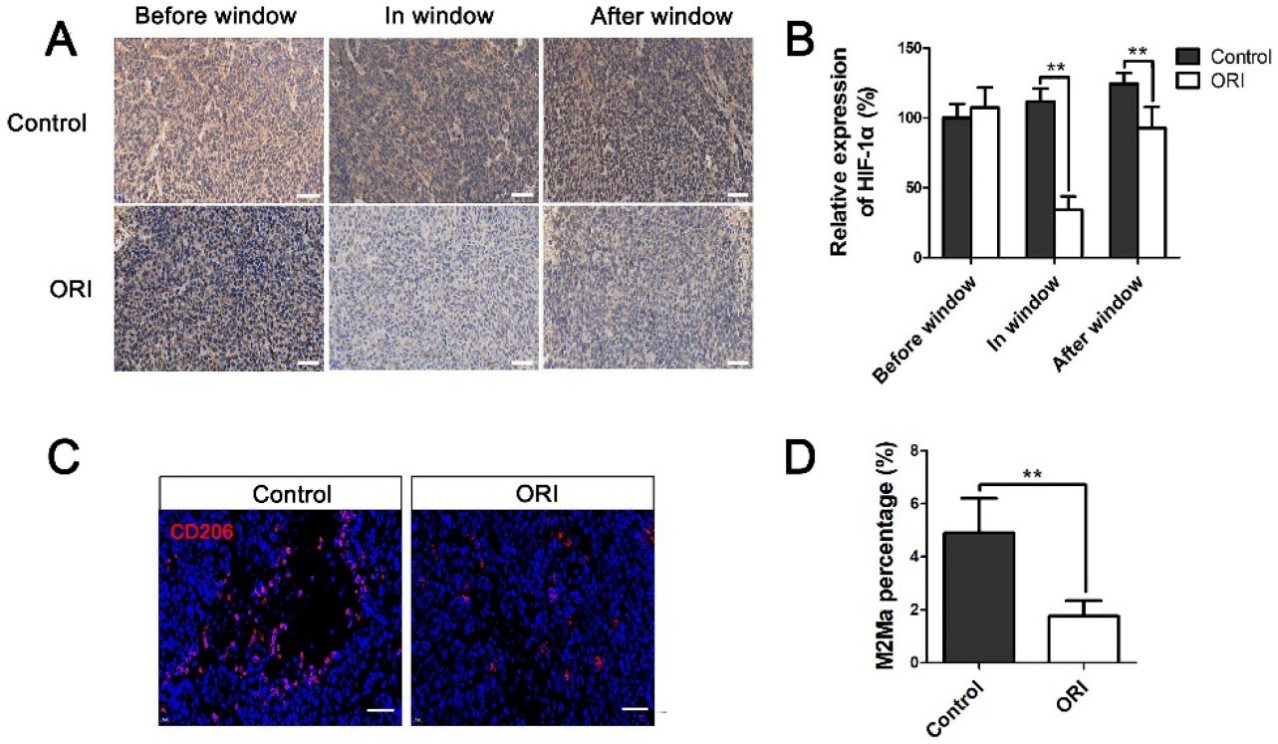
**ORI changed hypoxia and macrophage polarization of the TME in the time window of vascular normalization.** (A) Immunohistochemical staining for HIF-1α (×400, scale bar =50 µm). (B) The expression of HIF-1α was significantly downregulated within the normalization window (day 7) in the ORI group. (C) Representative images and quantification of M2 macrophages (stained by CD206) within tumor on day 7 (×400, scale bar =50 µm). *p < 0.05, **p < 0.01.

Considering that tumor vascular normalization alleviates hypoxia in the TME, we investigated the expression of HIF-1α. The results showed the expression of HIF-1α was significantly downregulated within the normalization window (day 7) in the ORI group (Figure 4A and 4B). Tumor-associated macrophages polarization plays a pivotal role in the vascular normalization. The results showed that M2-like macrophage was much lower in the ORI group than the Control group, which suggested the efficiency of macrophage reprogramming by ORI (Figure 4C and 4D). All these above results demonstrated that ORI could improve the vessel normalization and potently regulate the polarization of the tumor-associated macrophages away from M2 phenotype.

### The antitumor effect of ORI was associated with the JAK2/STAT3 signaling pathway

**Figure 5:**
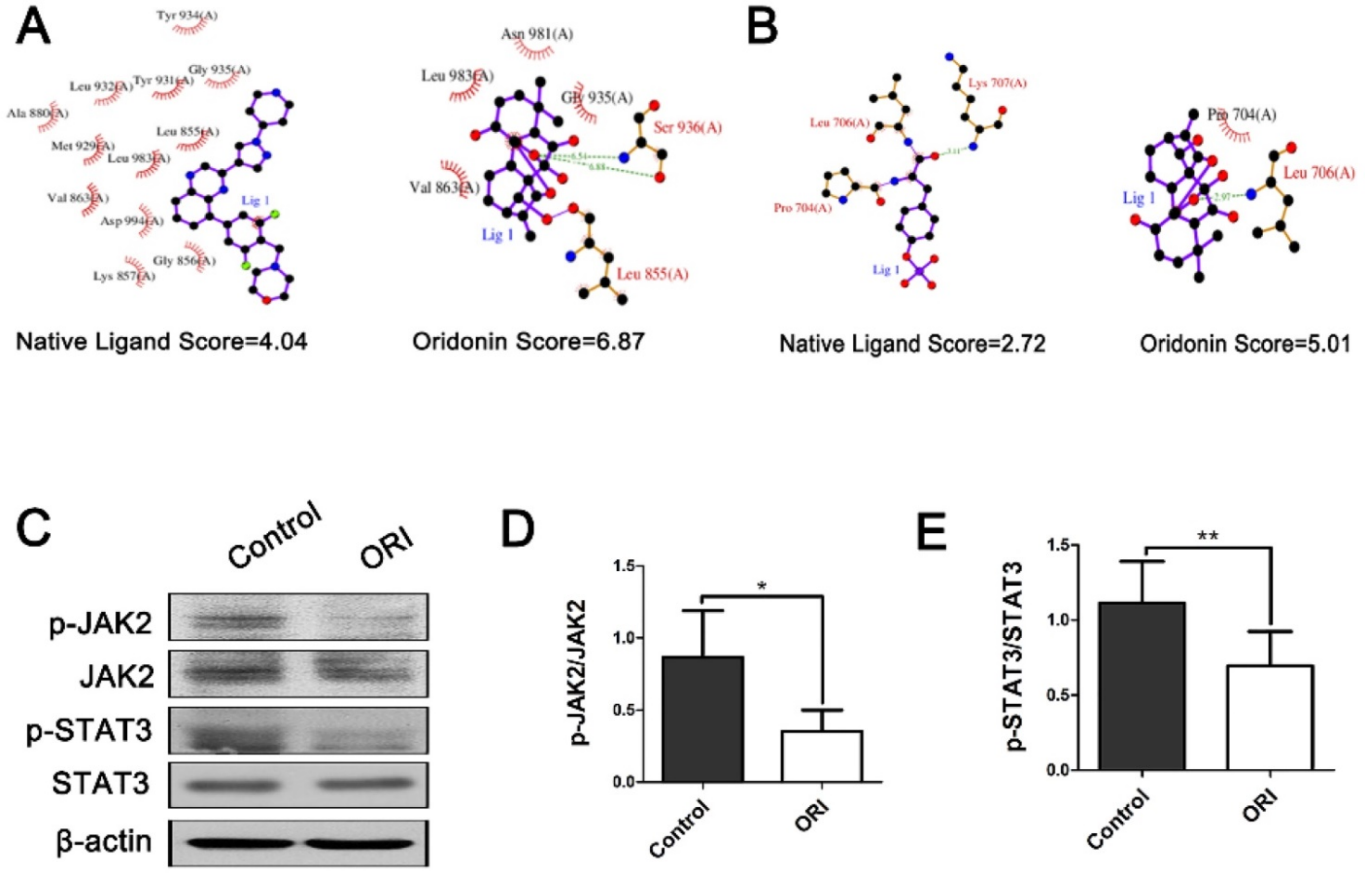
** The antitumor effect of ORI was associated with the JAK2/STAT3 signaling pathway.** Docking exercises of ORI binding to JAK2 (A) and STAT3 (B). (C) Representative Western blot expressions of p-JAK2, JAK2, p-STAT3 and STAT3. (D and E) The western blot analyses demonstrated declined phosphorylation of JAK2 and STAT3 in the ORI-treated group compared with controls. *p < 0.05, **p < 0.01.

Aberrant activation of JAK2/STAT3 signaling is frequently presented in colon tumors and implicated in the tumor vasculature reprogramming. We used molecular docking analyses to predict whether JAK2/STAT3 signaling pathway is the therapeutic targets of ORI. Therefore, we applied docking exercises of ORI binding to JAK2 and STAT3. The crystal structures of JAK2 and STAT3 were obtained from the Protein Data Bank (PDB ID of JAK2: 3KRR; PDB ID of STAT3: 1BG1). The docking exercises were conducted via systemsDock (http://systemsdock.unit.oist.jp/) 20. The results showed that the binding scores of ORI were 6.87 and 5.01, respectively. These docking scores were higher than that of the native ligand binding to JAK2 (4.04) and STAT3 (2.72) (Figure 5A and 5B). It suggested that JAK2/STAT3 signaling pathway might be one of the most important targets for ORI. Furthermore, we validated the prediction through the molecular biological method. The western blot analyses demonstrated declined phosphorylation of JAK2 and STAT3 in the ORI-treated group compared with Controls (Figure 5C, 5D and 5E). Our results indicated that ORI disrupted the JAK2/STAT3 signaling pathway in colon cancer. These discoveries collectively indicated the antitumor effect of ORI was closely associated with the JAK2/STAT3 signaling pathway.

## Discussion

The biological function of ORI on colon cancer has been investigated previously, but its role and the details concerning the underlying molecular mechanism still need to be further elucidated. Herein, we examined the anticancer effects of ORI on the subcutaneous HCT116- tumor-bearing mice. The results revealed that ORI inhibited the growth of tumor. It significantly reduced MVD, simultaneously increased the pericyte coverage rate of tumor vessels. Additionally, ORI-induced macrophage reprogramming. These anticancer effects of ORI may be partly mediated by suppressing JAK2/STAT3 signaling pathway.

Tumor angiogenesis, driven by an imbalance between pro- and anti-angiogenic signaling, is considered as one of the hallmarks of cancers 7. Counter-balance the pro- and anti-angiogenic factors toward equilibrium could normalize tumor vasculature. VEGF and bFGF are regarded as the most important pro-angiogenic growth factors. They participate in almost all steps in the angiogenesis process 21. Endostatin and angiostatin are vital anti-angiogenic factors 22, 23, these factors can inhibit endothelial cell proliferation, migration, microvessel assembly, and *in vivo* suppression of new blood vessel formation. In the present study, ORI showed its capability to restore the balance between pro- and anti-angiogenic factors.

Tumor blood vessels are tortuous and dilated, with aberrant endothelium and poor pericytes coverage, this structure and the resulting abnormal TME pose a formidable barrier to the delivery and efficacy of cancer therapy 24. Furthermore, the application of antiangiogenic agents will cause tumor vessels degeneration, thereby leads to tumor hypoxia and decreases tumor blood perfusion. Hence, antiangiogenic therapy seems would be anticipated to render many chemoradiotherapy and immunotherapy schedules less effective. However, paradoxically, accumulated evidence suggest that antiangiogenic agents augment rather than weaken the anticancer effects of radiation or chemotherapy 12,13,14. Fortunately, the proposition of the new concept that antiangiogenic therapy transiently normalizes abnormal tumor vasculature both structurally and functionally which providing a window of opportunity for enhancing the efficacy of chemotherapy or radiotherapy solves this contradiction well 15. Therefore, the therapeutic strategy has dual effects that can simultaneously inhibit tumor angiogenesis and timely induce vessel normalization is expected to be found. In the present study, we found that ORI significantly inhibited CD31^+^ microvessel density, which has obvious anti-angiogenesis effects. Simultaneously, Figure 3A and 3B showed that ORI intervention can increase perivascular cell coverage of colon cancer, and CD31^+^/α-SMA^+^ double immunofluorescent labeling was dramatically increased from days 5 to 10. The results suggested a time window for vessel normalization after ORI treatment. This window was short-lived. We can speculate that in the definite normalization time window, it would be possible to precisely handle the schedule of combination use of ORI with chemoradiotherapy or immunotherapy which will amplifying the potential anti-tumor effects. Tumor-associated macrophages play a significant role in promoting tumor progression. In malignant and advanced tumors, tumor-associated macrophages are biased toward the M2 phenotype. Recent studies have demonstrated that M2 macrophages depletion normalizes tumor blood vessels, which confirmed the important role of tumor-associated macrophages and their polarization away from M2 to M1 phenotype in tumor vessels normalization 25. In the present study, CD206^+^ M2-like macrophages were decreased by the ORI, which indicated that ORI-induced macrophage reprogramming exists for enhanced vessel normalization.

To further elucidate the mechanism underlying these phenomenons, SystemsDock (http://systemsdock.unit.oist.jp/), a web server for network pharmacology-based prediction and analysis was applied 20. Emerging evidence has suggested that the JAK2/STAT3 pathway participates in the regulation of angiogenesis 26, and blockade of the JAK2/STAT3 pathway could inhibit tumor growth. To explore whether JAK2/STAT3 signaling pathway is the therapeutic targets of ORI, we applied SystemsDock. These results revealed that JAK2/STAT3 signaling pathway might be one of the most important targets for ORI.

## Conclusion

In summary, we found that ORI significantly reduced MVD, induced macrophage reprogramming, simultaneously increased vascular mural cells coverage, improved hypoxia in a specific time period. The results have demonstrated that the antitumor effects of ORI might be mediated via vascular effects; it inhibited angiogenesis and inducing vessel normalization in colon cancer. These effects of ORI may be partly mediated by suppressing JAK2/STAT3 signaling pathway. Further, the formation of the “vascular normalization window” may provide a basis for ORI combination treatment with chemoradiotherapy or immunotherapy.

## Abbreviations

ORI: oridonin
TME: tumor microenvironment
JAK2: Janus kinase 2
STAT3: signal transducer and activator of transcription 3
TV: tumor volume
BSA: bovine serum albumin
MVD: microvessel density
VEGF: vascular endothelial growth factor
bFGF: basic fibroblast growth factor
